# RGB-D Mirror Segmentation with Reliability-Guided Residual Correction

**DOI:** 10.3390/s26123739

**Published:** 2026-06-11

**Authors:** Taehyeon Kim, Yong Ju Jung

**Affiliations:** School of Computing, Gachon University, Seongnam-si 13120, Republic of Korea; xogus9908@gachon.ac.kr

**Keywords:** mirror segmentation, RGB-D segmentation, dual-depth cues, deep learning

## Abstract

Mirror segmentation remains challenging because mirror regions often share appearance with the reflected scene, while sensor depth around mirrors is frequently missing, noisy, or geometrically inconsistent. Although recent RGB-based methods have achieved strong results by exploiting contextual and symmetry-aware cues, their ability to use geometric information reliably is still limited. In this paper, we propose a reliable RGB-D mirror segmentation framework built upon SATNet. Specifically, we extend the symmetry-aware baseline with a dedicated depth branch that injects hierarchical sensor-depth features into the multi-scale decoder, and we introduce a Reliability-Guided Residual Correction Module (RGRCM) for final prediction refinement. Instead of treating predicted depth as an independent modality branch, RGRCM internally constructs dual-depth evidence from sensor depth and monocular depth estimated by a pretrained Depth Anything v2 model, encoding raw depth observations, cross-depth discrepancies, validity cues, and local depth instability. The resulting evidence is used to guide uncertainty-aware residual correction only in regions where depth-driven refinement is likely to be beneficial. Experiments on the RGBD-Mirror benchmark show that the proposed method achieves 83.57 IoU, 0.899 $F_{\beta }$, 0.026 MAE, and 6.26 BER, outperforming existing RGB and RGB-D mirror segmentation methods.

## 1. Introduction

**Figure 1:**
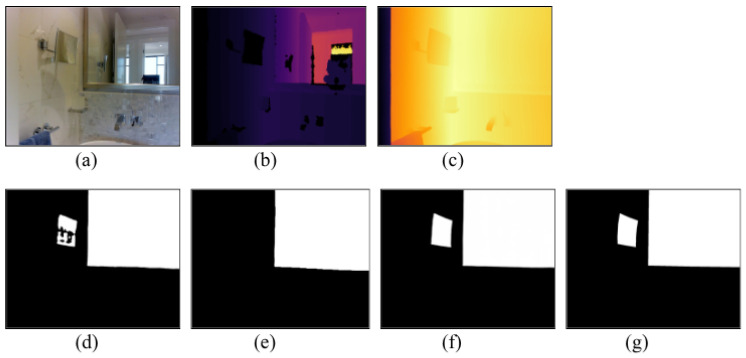
A motivating example on the RGBD-Mirror benchmark. (**a**) Input image, (**b**) sensor depth, (**c**) monocular predicted depth from Depth Anything v2, (**d**) SATNet, (**e**) UTLNet, (**f**) ours, and (**g**) ground truth. The sensor depth is severely corrupted around the mirror, while the predicted depth still provides a plausible geometric cue. As a result, existing methods may over-segment reflected content or miss the mirror region, whereas the proposed method better matches the ground truth.

Mirror segmentation aims to identify mirror regions at the pixel level. It is an important yet challenging problem in scene understanding because mirrors violate the common assumption that the appearance of an image region reflects the intrinsic property of a visible surface. Instead, mirror regions reflect surrounding objects and structures, often making them visually similar to the reflected scene itself. This ambiguity makes mirror segmentation difficult for models that rely mainly on local appearance or semantic context, and it also affects downstream tasks such as robotic navigation, scene parsing, augmented reality, and spatial perception [1,2]. As illustrated in Figure 1, mirror regions are difficult to segment not only because their appearance resembles the reflected scene, but also because the corresponding sensor depth is often missing, noisy, or geometrically inconsistent. This is a well-known limitation of depth sensors near reflective and transparent surfaces such as mirrors and glass [3,4]. In the example, the sensor depth map is severely corrupted around the mirror region, whereas the monocular predicted depth still preserves a plausible scene layout. Existing methods therefore tend to either over-segment reflected content or miss the mirror region entirely. This example highlights the central challenge of RGB-D mirror segmentation: depth is potentially useful, but it cannot be treated as uniformly reliable.

Recent progress in mirror segmentation has been largely driven by RGB-based methods. Early approaches exploited contextual contrast, boundary reasoning, and semantic cues to distinguish mirror interiors from their surroundings [1,5]. Subsequent methods introduced more intrinsic and structural cues, including visual chirality, semantic associations, and symmetry-aware reasoning [6,7,8]. In particular, SATNet demonstrated that the loose symmetry between the input image and its horizontally flipped counterpart provides a strong cue for mirror detection, establishing a powerful RGB baseline [8].

Despite these advances, RGB-only reasoning remains limited in challenging cases where appearance cues are ambiguous. In principle, depth can provide complementary geometric information for mirror segmentation. However, directly using sensor depth is nontrivial because mirror surfaces often produce missing, noisy, or inconsistent measurements. As a result, naive RGB-D fusion may introduce unreliable geometric cues rather than improve segmentation quality [2,9,10]. This observation suggests that the key issue is not simply how to add depth, but how to use depth reliably.

In this work, we propose a reliable RGB-D mirror segmentation framework built upon SATNet. Starting from the symmetry-aware RGB baseline, we first introduce a dedicated depth branch that extracts hierarchical geometric features from the sensor depth map and injects them into the multi-scale decoder. We then propose a *Reliability-Guided Residual Correction Module* (RGRCM), which performs uncertainty-aware residual correction on the final prediction. Rather than treating dual-depth information as an independent prediction pathway, RGRCM internally constructs discrepancy-aware depth evidence from the sensor depth and a monocular predicted depth generated by a pretrained Depth Anything v2 model [11]. This evidence is encoded by a Dual-Depth Evidence Block (DDEB), which aggregates raw dual-depth observations, cross-depth discrepancy cues, a joint-validity cue, and a local sensor-depth variance cue. The resulting evidence is then used to support selective residual correction only where such correction is likely to be beneficial.

This design is motivated by two observations. First, sensor depth remains useful for mirror segmentation whenever valid geometric measurements are available, and therefore a dedicated depth branch can complement symmetry-aware RGB reasoning. Second, in reflective regions, the relationship between sensor depth and monocular predicted depth (DA depth) often contains more useful information than either source alone. Accordingly, we do not use dual-depth evidence as a separate segmentation branch. Instead, we exploit it as an internal evidence representation within RGRCM, where it helps determine how depth-driven residual correction should be applied under ambiguous depth conditions.

Extensive experiments show that the proposed design is effective. The dedicated depth branch consistently strengthens the SATNet baseline, while RGRCM further improves the prediction through reliability-guided residual correction. The gains are particularly evident in terms of region overlap and balanced error, indicating that reliable depth utilization, rather than indiscriminate RGB-D fusion, is critical for robust mirror segmentation.

The main contributions of this work are summarized as follows:

- We extend SATNet to RGB-D mirror segmentation by introducing a dedicated depth branch that injects hierarchical sensor-depth features into the symmetry-aware decoder.
- We propose a Reliability-Guided Residual Correction Module (RGRCM), which internally constructs dual-depth evidence through a Dual-Depth Evidence Block (DDEB) and performs uncertainty-aware residual correction for final prediction refinement.
- We demonstrate through extensive experiments that the proposed framework improves mirror segmentation performance in a stable manner, especially in terms of region overlap and balanced error.

The rest of this paper is organized as follows. Section 2 reviews previous work related to mirror segmentation and reliability-aware RGB-D fusion. Section 3 describes the proposed method in detail, including the depth branch, the internal dual-depth evidence construction, the Reliability-Guided Residual Correction Module (RGRCM), and the loss function. Section 4 presents the experimental setup and quantitative results, including comparisons with state-of-the-art methods, ablation studies, and complexity analysis. Finally, Section 5 concludes the paper.

## 2. Related Work

### 2.1. Mirror Segmentation from RGB Images

Mirror segmentation has been actively studied from RGB images. MirrorNet introduced one of the earliest large-scale benchmarks for mirror segmentation and proposed contextual contrast modeling to distinguish mirror regions from surrounding content [1]. PMDNet further improved mirror localization by progressively learning contextual relations and explicitly refining mirror boundaries [5]. Later methods explored more intrinsic mirror cues. VCNet modeled visual chirality through a flipping–convolution–flipping transformation and introduced chirality-guided boundary reasoning [6], while semantic association-based methods leveraged the functional relation between mirrors and surrounding objects to improve robustness against distractors such as windows and doorways [7]. HetNet proposed multi-level heterogeneous learning to extract complementary mirror cues at different feature levels [12]. More recently, SATNet proposed a symmetry-aware transformer architecture that models the loose symmetry between the input image and its horizontally flipped counterpart, providing a strong RGB baseline for mirror detection [8].

### 2.2. RGB-D Mirror Segmentation

Compared with RGB-only approaches, RGB-D mirror segmentation seeks to exploit geometric cues to reduce appearance ambiguity. A representative early method in this direction is PDNet, which introduced the RGBD-Mirror dataset and showed that depth discontinuities and color-depth correlations can substantially improve mirror localization and boundary refinement [2]. More recent RGB-D mirror segmentation methods have focused on stronger multi-modal fusion, uncertainty modeling, and knowledge distillation. UTLNet introduced an uncertainty-aware transformer localization framework for RGB-depth mirror segmentation, explicitly modeling unreliable depth during localization and fusion [13]. Morphology-Guided Network (MGNet) further improved RGB-D mirror segmentation by incorporating morphology-aware structural guidance into a knowledge distillation framework [14]. ADRNet-S* proposed asymmetric depth registration and contrastive knowledge distillation for RGB-D mirror segmentation, emphasizing cross-modal alignment and depth-guided multimodal interaction [9]. NDANet-S* advanced this line by introducing neighborhood-level feature matching and demand-modal adaptive fusion within a distillation framework [15]. Recently, Kurohiji and Hachiya showed that inconsistency between sensor depth and predicted depth itself is a useful cue for mirror segmentation, and they proposed a depth inconsistency-based spatial-channel attention gate to emphasize informative reflective regions [16].

Our approach is distinct from depth inconsistency-based mirror segmentation methods such as that of Kurohiji and Hachiya [16], who employ the inconsistency between sensor and predicted depth as a spatial-channel attention cue during feature fusion. The key difference lies in where and how the dual-depth cue is exploited: rather than acting as an attention modulation distributed across the decoder, our dual-depth evidence is consumed at the final prediction stage to guide explicit residual correction. Furthermore, the proposed safe loss regularization explicitly penalizes unnecessary correction on pixels where the base prediction is already confident and correct, a conservative correction principle that is not present in prior reliability-aware fusion or depth inconsistency attention designs.

These studies consistently show that depth is a valuable cue for mirror segmentation. At the same time, they also reveal a common challenge: the benefit of depth depends strongly on how geometric information is represented, aligned, and fused, especially when depth measurements are missing or unreliable around reflective surfaces.

### 2.3. Reliability-Aware RGB-D Fusion and Dual-Depth Cues

The broader RGB-D segmentation literature has repeatedly shown that depth should not be treated as uniformly reliable. ACNet selectively gathers complementary RGB and depth information through attention-based fusion [17]. SA-Gate reduces the influence of noisy depth measurements through bi-directional cross-modal gating [18]. UCTNet further models depth uncertainty explicitly and uses uncertainty-aware cross-modal interaction for robust RGB-D semantic segmentation [10]. More broadly, handling unreliable or heterogeneous depth cues is a shared challenge across multi-modal vision tasks, including RGB-D salient object detection [19,20,21] and depth estimation [22,23,24]. In parallel, monocular depth estimation has improved significantly in recent years. Depth Anything v2 provides fine-grained and robust depth predictions with strong generalization ability, making predicted depth a practical complementary cue when sensor depth is degraded [11]. Motivated by these observations, our method does not use predicted depth as an independent modality branch. Instead, it combines sensor depth and predicted depth to internally construct dual-depth evidence inside the proposed RGRCM and uses this evidence to guide selective residual correction.

In summary, existing RGB mirror segmentation methods mainly exploit contextual contrast, semantic relations, chirality, or symmetry-aware reasoning, whereas RGB-D approaches demonstrate the value of geometry but remain sensitive to unreliable depth and suboptimal fusion. Our method differs in two main aspects: first, it extends the strong SATNet baseline to the RGB-D setting through a dedicated depth branch; second, it introduces a reliability-guided residual correction design in which dual-depth evidence is internally constructed and used for the uncertainty-aware correction of the final prediction.

## 3. Method

**Figure 2:**
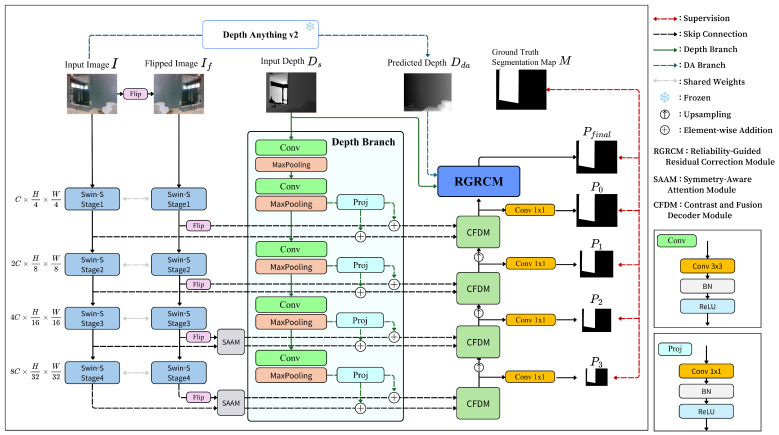
Overall architecture of the proposed method.

**Figure 3:**
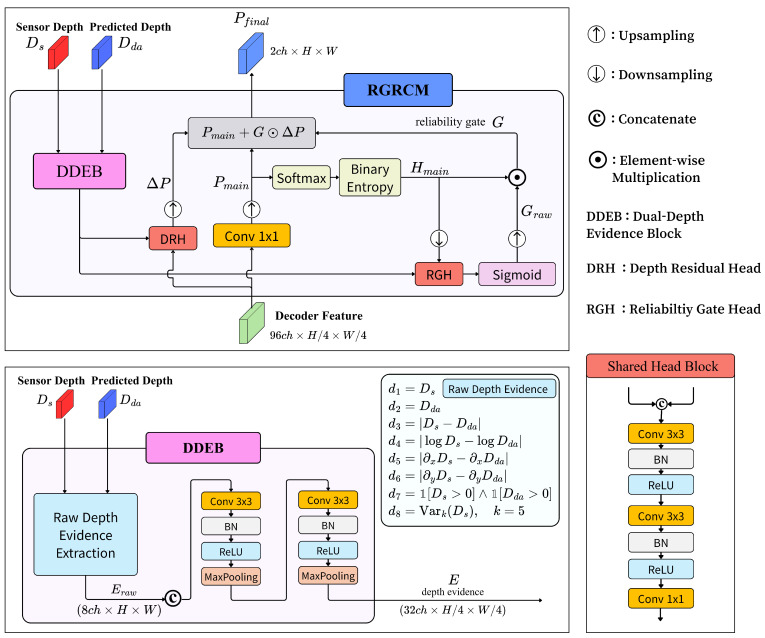
Structure of the reliability-guided residual correction module (RGRCM).

This section presents the proposed RGB-D mirror segmentation framework. As shown in Figure 2, the network is built upon SATNet and augments the original symmetry-aware RGB pipeline with two key additions: a dedicated depth branch for encoding sensor-depth geometry, and a *Reliability-Guided Residual Correction Module* (RGRCM) for final prediction refinement. A key design choice is that dual-depth information is not treated as an independent prediction branch. Instead, it is internally constructed inside RGRCM as discrepancy-aware evidence and is used to support reliability-guided residual correction. The detailed structure of RGRCM, including the internal *Dual-Depth Evidence Block* (DDEB), is shown in Figure 3.

### 3.1. Overall Architecture

Let $I\in R^{3\times H\times W}$ denote the input RGB image, $D_{s}\in R^{1\times H\times W}$ the sensor depth map, and $M\in {\left\{0,1\right\}}^{H\times W}$ the ground-truth mirror mask. Following SATNet, we construct a horizontally flipped image

(1) $$I_{f}=F\left(I\right),$$

where F(·) denotes horizontal flipping.

The original image *I* and the flipped image $I_{f}$ are then processed by two weight-sharing Swin-S encoders. Denoting the shared RGB encoder by $E_{rgb}$, the resulting multi-scale feature pyramids are

(2) $${\left\{X_{k}\right\}}_{k=0}^{3}=E_{rgb}\left(I\right),\;{\left\{X_{k}^{f}\right\}}_{k=0}^{3}=E_{rgb}\left(I_{f}\right),$$

where k=0,1,2,3 correspond to feature resolutions H/4, H/8, H/16, and H/32, respectively. The flipped-stream features are spatially realigned by inverse flipping:

(3) $${\bar{X}}_{k}^{f}=F^{-1}\left(X_{k}^{f}\right).$$

Following SATNet, the symmetry-aware attention module (SAAM) models the interaction between the main-stream features and the realigned flipped-stream features, and the contrast and fusion decoder module (CFDM), which incorporates efficient channel attention [25], progressively decodes the fused RGB representations to produce hierarchical decoder features and auxiliary predictions [8]. As shown in Figure 2, the decoder outputs four auxiliary predictions, denoted by $P_{3}$, $P_{2}$, $P_{1}$, and $P_{0}$, and the top decoder feature is further refined by RGRCM to generate the final output $P_{final}$.

In parallel, the sensor depth map $D_{s}$ is processed by a dedicated depth branch that extracts multi-scale geometric features aligned with the decoder hierarchy. These projected depth features are injected into the decoder through element-wise addition, enabling the symmetry-aware RGB decoder to exploit explicit geometry throughout the decoding process.

To provide complementary depth cues, we additionally obtain a monocular predicted depth map from a pretrained and frozen Depth Anything v2 model:

(4) $$D_{da}=\Phi_{DA}\left(I\right),$$

where $\Phi_{DA}\left(\cdot \right)$ denotes the Depth Anything v2 estimator. The predicted depth $D_{da}$ is not directly fused into the decoder. Instead, it is used only inside RGRCM, where it is combined with the sensor depth $D_{s}$ to construct dual-depth evidence for final-stage residual correction.

Let $F_{top}\in R^{96\times H/4\times W/4}$ denote the top decoder feature from CFDM, as shown in Figure 3. The final mirror prediction is obtained by the proposed RGRCM:

(5) $$P_{final}=\mathrm{RGRCM}\left(F_{top},D_{s},D_{da}\right),$$

where $P_{final}\in R^{2\times H\times W}$ denotes the final two-channel logits.

### 3.2. Depth Branch

The depth branch is designed to encode the sensor depth map into hierarchical geometric features that are spatially aligned with the SATNet decoder. While RGB appearance is often ambiguous in reflective regions, valid depth measurements provide direct structural cues that are useful for mirror localization and boundary recovery.

As shown in Figure 2, the depth branch consists of a sequence of convolutional blocks. Each block is composed of a 3×3 convolution, batch normalization, and ReLU activation, followed by max-pooling for downsampling. Let $B^{\left(t\right)}$ denote the intermediate feature at stage *t*. The depth encoding process is written as

(6) $$B^{\left(0\right)}=D_{s},\;B^{\left(t\right)}=\mathrm{Pool}\;\left(\phi^{\left(t\right)}\left(B^{\left(t-1\right)}\right)\right),\;t=1,\ldots ,T,$$

where $\phi^{\left(t\right)}\left(\cdot \right)$ denotes a Conv-BN-ReLU block and Pool(·) denotes max-pooling.

To match the decoder hierarchy, a projection block is attached to each decoding scale. Let $G_{k}$ denote the projected depth feature at level *k*. Then

(7) $$G_{k}=\Pi_{k}\left(B_{k}\right),\;k\in \left\{0,1,2,3\right\},$$

where $\Pi_{k}\left(\cdot \right)$ denotes a 1×1 Conv-BN-ReLU projection block, and $G_{k}$ has the same spatial resolution and channel dimension as the corresponding decoder skip feature.

At each decoder stage, the projected depth feature is injected into the RGB skip features through element-wise addition. Let $S_{k}^{\left(1\right)}$ and $S_{k}^{\left(2\right)}$ denote the two skip tensors used by the CFDM block at level *k*. The depth-enhanced skip features are defined as

(8) $${\tilde{S}}_{k}^{\left(1\right)}=S_{k}^{\left(1\right)}+G_{k},\;{\tilde{S}}_{k}^{\left(2\right)}=S_{k}^{\left(2\right)}+G_{k}.$$

This design preserves the original symmetry-aware decoder topology of SATNet while enabling the network to exploit sensor-depth geometry throughout multi-scale decoding. Importantly, the depth branch uses only sensor depth and focuses on hierarchical geometric encoding, whereas the dual-depth representation is reserved for the final reliability-guided correction stage.

### 3.3. Reliability-Guided Residual Correction Module

The proposed RGRCM performs final-stage residual correction using both prediction uncertainty and internally constructed dual-depth evidence. Its purpose is to avoid the indiscriminate use of depth and instead apply depth-driven correction only where such correction is needed and likely to be reliable. As shown in Figure 3, RGRCM consists of three functional stages: 1) dual-depth evidence construction through DDEB, 2) main prediction and uncertainty estimation, and 3) reliability-guided residual correction.

#### 3.3.1. Dual-Depth Evidence Block

The purpose of DDEB is to construct discrepancy-aware depth evidence from the sensor depth and the monocular predicted depth. Rather than acting as an independent branch, DDEB provides an internal evidence representation for the subsequent residual correction and reliability gating inside RGRCM.

Given the sensor depth $D_{s}$ and the predicted depth $D_{da}$, we first resize both maps to the input resolution. We do not apply per-image depth normalization or scale-shift alignment before constructing the evidence channels. Although $D_{s}$ and $D_{da}$ are not necessarily metrically aligned, the proposed DDEB uses their raw preprocessed responses as learnable inconsistency cues rather than calibrated metric differences. This preserves sensor-depth failure patterns, local instability, and the native response distribution of the predicted depth. We define the following raw depth evidence channels:

(9) $$\begin{array}{cccccc} e_{1} & =D_{s},\;\;\;\;\;\;\;\;\;\;\;\;\;\;\;\;\;\;\;\;\;\;\;\;\;\;\;\;\;\;\;\;\;\;\;\;\;\;\;\;\;\;\;\;\;\;\;\;\;\; & e_{2} & =D_{da},\;\;\;\;\;\;\;\;\;\;\;\;\;\;\;\;\;\;\;\; & e_{3} & =|D_{s}-D_{da}|, \end{array}$$

(10) $$\begin{array}{cccccc} e_{4} & =\left|\log\left(D_{s}+\epsilon \right)-\log\left(D_{da}+\epsilon \right)\right|, & e_{5} & =|\partial_{x}D_{s}-\partial_{x}D_{da}|, & e_{6} & =|\partial_{y}D_{s}-\partial_{y}D_{da}|, \end{array}$$

(11) $$\begin{array}{cccc} e_{7} & =1\left[D_{s}>0\right]\wedge 1\left[D_{da}>0\right],\;\;\;\;\;\;\;\;\;\;\;\;\;\;\; & e_{8} & ={\mathrm{Var}}_{k}\left(D_{s}\right),\;k=5, \end{array}$$

where ϵ is a small constant for numerical stability, $\partial_{x}$ and $\partial_{y}$ denote horizontal and vertical finite-difference operators, ∧ denotes logical conjunction, and ${\mathrm{Var}}_{k}\left(D_{s}\right)$ denotes the local variance of the sensor depth computed over a k×k patch. In our implementation, k=5. The first two channels preserve the original depth observations, $e_{3}$ and $e_{4}$ encode raw cross-depth inconsistency in linear and logarithmic spaces, $e_{5}$ and $e_{6}$ capture gradient inconsistency, and $e_{7}$ provides a joint-validity cue for reliable cross-depth comparison. Since the predicted depth is dense in practice, this term primarily acts as a validity cue for cross-depth comparison in regions where sensor depth is available. $e_{8}$ encodes the local instability of the sensor depth that frequently occurs around reflective surfaces and invalid depth regions.

The raw depth evidence tensor is then formed by channel-wise concatenation:

(12) $$E_{raw}=\left[e_{1};e_{2};e_{3};e_{4};e_{5};e_{6};e_{7};e_{8}\right]\in R^{8\times H\times W}.$$

As shown in Figure 3, $E_{raw}$ is subsequently encoded by a shallow evidence encoder $\Psi_{e}\left(\cdot \right)$, composed of stacked Conv-BN-ReLU-MaxPooling blocks:

(13) $$E=\Psi_{e}\left(E_{raw}\right),\;E\in R^{C_{e}\times H/4\times W/4},$$

where $C_{e}$ denotes the channel dimension of the encoded evidence feature.

DDEB therefore converts raw dual-depth observations into a compact discrepancy-aware representation that can be used internally by RGRCM. This design allows the network to exploit the relationship between the two depth sources without introducing a separate segmentation pathway.

#### 3.3.2. Main Prediction and Uncertainty Estimation

Given the top decoder feature $F_{top}$, RGRCM first computes a main prediction branch:

(14) $$P_{main}=\mathrm{Up}\;\left({\mathrm{Conv}}_{1\times 1}\left(F_{top}\right)\right),$$

where $P_{main}\in R^{2\times H\times W}$ and Up(·) denotes bilinear upsampling to the input resolution. This branch corresponds to the baseline segmentation logits before reliability-guided correction.

We then obtain the posterior probability map by softmax:

(15) $$Q=\mathrm{Softmax}(P_{main}),$$

where $Q\in {\left[0,1\right]}^{2\times H\times W}$. Based on this, we compute the normalized binary entropy map

(16) $$H_{main}=-\frac{1}{\log2}\sum_{c=1}^{2}Q_{c}\log\left(Q_{c}+\epsilon \right),$$

where $H_{main}\in {\left[0,1\right]}^{1\times H\times W}$. A larger value of $H_{main}$ indicates greater uncertainty in the main prediction and therefore a stronger need for correction.

This uncertainty estimate plays a central role in RGRCM. Rather than correcting all pixels uniformly, the module uses the entropy map to identify ambiguous regions where depth-driven residual refinement is more likely to be beneficial.

#### 3.3.3. Reliability-Guided Residual Correction

The final stage of RGRCM performs reliability-guided residual correction based on the top decoder feature $F_{top}$, the encoded depth evidence E, and the uncertainty map $H_{main}$.

First, a *Depth Residual Head* (DRH) predicts a depth-driven residual correction:

(17) $$\Delta P=\mathrm{Up}\;\left(\mathrm{DRH}\;\left([F_{top};E]\right)\right),$$

where $\Delta P\in R^{2\times H\times W}$ and [·;·] denotes channel-wise concatenation. As shown in Figure 3, DRH is implemented using the shared head architecture composed of two 3×3 Conv-BN-ReLU layers followed by a final 1×1 convolution.

Next, a *Reliability Gate Head* (RGH) estimates a learned gate conditioned on the encoded evidence and the uncertainty map. Specifically, the encoded evidence E and the downsampled uncertainty map $\mathrm{Down}(H_{main})$ are concatenated and fed into RGH:

(18) $$G_{raw}=\mathrm{Up}\;\left(\sigma \;\left(\mathrm{RGH}\;\left([E;\mathrm{Down}\left(H_{main}\right)]\right)\right)\right),$$

where $G_{raw}\in {\left[0,1\right]}^{1\times H\times W}$ and σ(·) denotes the sigmoid function. RGH has the same shared head architecture as DRH but uses independent parameters.

The final gate is obtained by modulating the learned gate with the uncertainty map:

(19) $$G=H_{main}⊙G_{raw},$$

where ⊙ denotes element-wise multiplication.

Finally, the corrected output is given by

(20) $$P_{final}=P_{main}+G⊙\Delta P,$$

where the single-channel gate *G* is broadcast along the class dimension.

This formulation has a clear interpretation. The main branch first provides a stable baseline prediction. RGRCM then constructs dual-depth evidence internally through DDEB and predicts a residual correction through DRH, but it applies that correction only where the current prediction is uncertain and where RGH judges the correction to be reliable. In this way, RGRCM suppresses harmful depth-driven correction in confident regions while selectively refining ambiguous mirror regions.

### 3.4. Loss Function

The proposed network is trained with three complementary objectives: a baseline multi-scale supervision term inherited from SATNet, a final prediction loss for the corrected output, and a safe regularization term for the proposed RGRCM. The overall objective is defined as

(21) $$L=L_{\mathrm{final}}+L_{\mathrm{base}}+\lambda L_{\mathrm{safe}},$$

where λ controls the contribution of the safe regularization term. In all experiments, we set λ=0.1.

Following SATNet [8], we apply deep supervision to the four decoder predictions ${\left\{P_{i}\right\}}_{i=0}^{3}$. The baseline loss is defined as

(22) $$L_{\mathrm{base}}=\sum_{i=0}^{3}w_{i}L_{\mathrm{ce}}\left(P_{i},M\right),$$

where $w_{i}$ is the scale-dependent weight for the *i*-th prediction map, and $L_{\mathrm{ce}}\left(\cdot ,\cdot \right)$ denotes the pixel-wise cross-entropy loss. Following the SATNet [8] setting, we use [1.25, 1.25, 1.0, 1.5] for [$w_{0}$, $w_{1}$, $w_{2}$, $w_{3}$]. This term preserves the original multi-scale supervision strategy of SATNet [8] and stabilizes the learning of the symmetry-aware decoder.

In addition to the auxiliary supervision, we directly supervise the final corrected prediction $P_{final}$ produced by RGRCM:

(23) $$L_{\mathrm{final}}=L_{\mathrm{ce}}\left(P_{final},M\right).$$

This term ensures that the final output after reliability-guided residual correction is optimized directly for mirror segmentation.

For a prediction *P*, the cross-entropy loss is defined as

(24) $$L_{\mathrm{ce}}\left(P,M\right)=-\frac{1}{N}\sum_{j=1}^{N}\sum_{c\in \{0,1\}}y_{j,c}\log p_{j,c},$$

where N=H×W, $p_{j,c}$ is the softmax probability of class *c* at pixel *j*, and $y_{j,c}$ is the corresponding one-hot ground-truth label.

To explicitly encourage RGRCM to avoid unnecessary correction on already reliable predictions, we introduce a safe regularization term:

(25) $$L_{\mathrm{safe}}=\frac{1}{N}\sum_{j=1}^{N}e^{-\alpha H_{j}}\cdot c_{j}\cdot {∥G_{j}⊙\Delta P_{j}∥}_{1},$$

where α controls the sensitivity to uncertainty, $H_{j}$ is the uncertainty at pixel *j*, $c_{j}$ is the agreement coefficient between the main prediction and the ground truth, $G_{j}$ is the reliability gate, and $\Delta P_{j}$ is the residual correction predicted by RGRCM. Since $G_{j}$ is a one-channel gate, it is broadcast along the class dimension when multiplied with $\Delta P_{j}$. In our experiments, we set α=5.0.

In our framework, the main prediction corresponds to the pre-correction output $P_{main}$, which is identical to the top-scale prediction $P_{0}$. Let

(26) $$Q=\mathrm{Softmax}(P_{main}),$$

where $Q\in {\left[0,1\right]}^{2\times H\times W}$. We then compute the normalized binary entropy as

(27) $$H_{j}=-\frac{1}{\log2}\sum_{c=1}^{2}Q_{j,c}\log\left(Q_{j,c}+\epsilon \right),$$

where $Q_{j,c}$ is the posterior probability of class *c* at pixel *j*, and ϵ is a small constant for numerical stability.

The agreement coefficient $c_{j}$ is defined as

(28) $$c_{j}=\sum_{c=1}^{2}y_{j,c}Q_{j,c},$$

which corresponds to the posterior probability assigned by the main prediction to the ground-truth class at pixel *j*. Therefore, $c_{j}$ becomes large when the main prediction already agrees well with the ground truth.

The safe loss in Equation (25) penalizes large residual corrections on pixels where the main prediction is already correct and confident. Specifically, the factor $e^{-\alpha H_{j}}$ imposes a stronger penalty when the uncertainty is low, while the coefficient $c_{j}$ further emphasizes pixels whose main prediction already agrees well with the ground truth. As a result, $L_{\mathrm{safe}}$ encourages RGRCM to behave conservatively on easy and already-correct pixels, while allowing larger corrections on ambiguous or erroneous regions. This is consistent with the design goal of reliability-guided residual correction, namely, to suppress unnecessary modification of stable predictions and focus correction on difficult mirror regions.

## 4. Experiments and Results

This section evaluates the proposed method from multiple perspectives, including state-of-the-art comparison, qualitative analysis, component ablation, uncertainty-map visualization, statistical stability, robustness to sensor-depth corruption, depth-source analysis, safe loss and hyperparameter studies, computational complexity, and failure-case analysis.

### 4.1. Experimental Setup

#### Dataset and Evaluation Metrics

We evaluate the proposed method on the RGBD-Mirror benchmark [2], following the standard evaluation protocol used in prior work. RGBD-Mirror contains 3049 RGB-D image triplets, where each sample consists of an RGB image, a depth map, and a corresponding ground-truth mirror mask. The dataset is split into 2000 training images and 1049 testing images. Performance is measured using four widely adopted metrics, namely, intersection over union (IoU), $F_{\beta }$, mean absolute error (MAE), and balanced error rate (BER).

Let $\hat{M}\in {\left[0,1\right]}^{H\times W}$ denote the predicted foreground probability map and $M\in {\left\{0,1\right\}}^{H\times W}$ denote the ground-truth binary mirror mask. Let $\tilde{M}$ denote the binarized prediction obtained from $\hat{M}$, and let TP, TN, FP, and FN be the corresponding numbers of true positives, true negatives, false positives, and false negatives.

The IoU is defined as

(29) $$\mathrm{IoU}=\frac{TP}{TP+FP+FN}.$$

The $F_{\beta }$ score is defined as

(30) $$F_{\beta }=\frac{(1+\beta^{2})\;\mathrm{Precision}\cdot \mathrm{Recall}}{\beta^{2}\;\mathrm{Precision}+\mathrm{Recall}},$$

where

(31) $$\mathrm{Precision}=\frac{TP}{TP+FP},\;\mathrm{Recall}=\frac{TP}{TP+FN}.$$

The MAE is computed on the continuous prediction map as

(32) $$\mathrm{MAE}=\frac{1}{N}\sum_{i=1}^{N}\left|{\hat{m}}_{i}-m_{i}\right|,$$

where N=H×W, and ${\hat{m}}_{i}$ and $m_{i}$ denote the predicted foreground probability and the ground-truth binary label at pixel *i*, respectively.

The BER is defined as

(33) $$\mathrm{BER}=\left(1-\frac{1}{2}\left(\frac{TP}{TP+FN}+\frac{TN}{TN+FP}\right)\right)\times 100.$$

Higher IoU and $F_{\beta }$ indicate better segmentation performance, while lower MAE and BER indicate more accurate and balanced predictions. In all experiments, we set $\beta^{2}=0.3$ for $F_{\beta }$, and use a fixed binarization threshold of 0.5 to obtain the binary prediction map. All threshold-based metrics reported in this paper are computed under this setting.

### 4.2. Implementation Details

We adopt Swin-S [26] pretrained on ImageNet-1K [27] as the RGB backbone encoder. All experiments are conducted using input RGB images and depth maps resized to 512×512. The monocular predicted depth is obtained from a pretrained Depth Anything v2 [11] model, which is kept frozen during training. The remaining network, including the RGB backbone, the depth branch, the decoder, and RGRCM, is trained end-to-end using the loss function described in Section 3.4. Following Section 3.4, the multi-scale weights are set to [1.25,1.25,1.0,1.5], while the coefficients of the safe loss are set to λ=0.1 and α=5.0.

We train the network for 200 epochs using AdamW with $\beta_{1}=0.9$, $\beta_{2}=0.999$, and weight decay =0.01. The learning rate is set to $3\times 10^{-4}$. A PolynomialLR scheduler is adopted during training. The batch size is set to 8, and all experiments are conducted on a single NVIDIA GeForce RTX 5090 GPU.

Sensor depth maps are loaded as single-channel depth images and converted to the [0,1] range according to the stored 8-bit depth scale. No explicit masking, interpolation, hole-filling, or inpainting is applied to invalid or missing depth pixels at the preprocessing stage. Zero-valued or missing depth pixels are retained as zero values because missingness itself provides useful evidence near reflective surfaces. DA depth maps generated by Depth Anything v2 undergo the same [0,1] normalization. Instead of relying on hand-crafted preprocessing heuristics, we provide raw depth observations to the network and rely on the internal evidence mechanisms described in Section 3.3.1—in particular, the joint-validity channel $e_{7}$ and the local-variance channel $e_{8}$—to identify and handle unreliable measurements during inference.

During training, we apply several data augmentation strategies to improve generalization. Specifically, random cropping is applied with a crop size of up to 25% of the image area, random horizontal flipping is applied with probability 0.5, and random brightness and contrast jittering within ±0.2 is applied with probability 0.5. In addition, random Gaussian blur with a kernel size 3 or 5 is applied with probability 0.3, and random Gaussian noise with standard deviation σ=5 is applied with probability 0.3. After augmentation, all RGB images and depth maps are resized to 512×512, and the RGB images are normalized using the ImageNet mean and standard deviation. During testing, only resizing and RGB normalization are applied.

### 4.3. Comparison with State-of-the-Art Methods

Table 1 compares the proposed method with representative RGB mirror segmentation methods and RGB-D mirror segmentation methods on the RGBD-Mirror benchmark [2]. Specifically, the compared RGB mirror methods include VCNet [6], SATNet [8], CSFwinformer-B [28], DPRNet [29], S2MD [30], and SAMirror [31], while the compared RGB-D mirror methods include PDNet [2], SANet [7], NDANet-S* [15], MGNet-S* [14], UTLNet [13], ADRNet-S* [9], and Kurohiji and Hachiya [16]. Except for Kurohiji and Hachiya, all threshold-based metrics are evaluated using the same fixed binarization threshold of 0.5. Since Kurohiji and Hachiya report the mean values of the top five runs selected from ten repeated trials in their original paper, we include their reported mean values for reference only. These methods cover both strong RGB baselines and recent RGB-D approaches, providing a comprehensive benchmark for evaluating the effectiveness of the proposed depth branch and RGRCM. For quantitative comparison, SATNet (our baseline) was retrained using the official codebase under our experimental setting. For all other competing methods, performance numbers are cited directly from their respective original publications. Note that we group SAMirror [31] with RGB methods because its main segmentation backbone is RGB-driven while predicted depth is used as an auxiliary cue.

The proposed method achieves the best overall performance, reaching 83.57 IoU, 0.899 $F_{\beta }$, 0.026 MAE, and 6.26 BER. In particular, our method outperforms all compared methods in IoU, $F_{\beta }$, and BER, while also matching the best MAE.

Compared with the RGB baseline SATNet [8], the proposed method improves IoU from 80.69 to 83.57, $F_{\beta }$ from 0.877 to 0.899, and BER from 7.33 to 6.26. Compared with the strongest RGB-D competitor ADRNet-S* [9], our method further improves IoU by 1.36 points, $F_{\beta }$ by 0.028, and BER by 0.76 points. These results verify that the proposed depth branch and RGRCM provide more effective and more reliable depth utilization than conventional RGB-only reasoning or direct RGB-D fusion.

**Figure 4:**
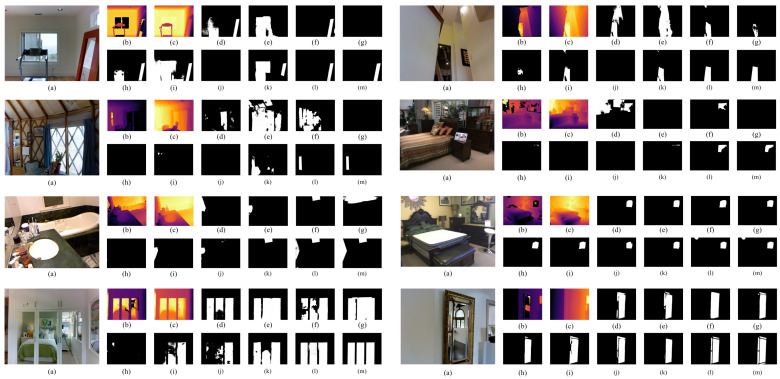
Qualitative comparison on the RGBD-Mirror benchmark. Columns from left to right show (**a**) input image, (**b**) sensor depth, (**c**) monocular predicted depth from Depth Anything v2, (**d**) PDNet, (**e**) SANet, (**f**) VCNet, (**g**) SATNet, (**h**) CSFwinformer-B, (**i**) DPRNet, (**j**) S2MD, (**k**) UTLNet, (**l**) ours, and (**m**) ground-truth mask.

Figure 4 presents representative qualitative comparisons between the proposed method and prior RGB and RGB-D mirror segmentation methods. Across all examples, the sensor depth is often incomplete, noisy, or unreliable around reflective regions, whereas the predicted depth provides a more plausible global scene layout. This observation supports the design motivation of the proposed framework, which uses sensor depth as the primary geometric cue in the depth branch and exploits the predicted depth only as complementary evidence inside RGRCM. Note that, for qualitative comparison in Figure 4, we used officially released prediction maps from methods with publicly available code. SANet predictions were generated using the authors’ released model weights.

### 4.4. Qualitative Results

In the first row, window-like structures are easily confused with mirrors because of their similar rectangular shape and reflective appearance. Several competing methods produce noticeable false positives or over-segment the ambiguous region, whereas the proposed method better suppresses non-mirror structures and produces a mask that is more consistent with the ground truth. This suggests that the proposed reliability-guided correction helps avoid erroneous depth-driven updates in visually confusing regions.

The second to fourth rows show mirrors that are difficult to recognize due to weak appearance cues, narrow shapes, or challenging scene context. In these cases, several existing methods either miss the mirror entirely or predict incomplete masks. By contrast, the proposed method better recovers the correct mirror extent, especially when the sensor depth alone is insufficient but the dual-depth evidence still provides useful complementary guidance.

The fifth and sixth rows contain scenes with two mirrors of different sizes. These examples are challenging because small mirrors are easily missed while large mirrors may dominate the prediction. The proposed method is able to preserve both mirror regions more reliably, indicating that the combination of hierarchical depth features and reliability-guided residual correction improves scale robustness.

The last two rows show scenes containing multiple mirrors. Existing methods often miss one of the mirror regions, produce fragmented masks, or fail to localize all reflective areas consistently. In contrast, the proposed method detects multiple mirror regions more completely and yields predictions that are closer to the ground truth. Overall, the qualitative results demonstrate that the proposed framework is particularly effective in ambiguous scenes where naive use of depth is unreliable and selective correction is necessary.

### 4.5. Ablation Study

We conduct ablation studies to analyze the contribution of the proposed architectural components, the roles of different depth inputs and the proposed dual-depth evidence, and the effect of the safe loss term. Unless otherwise specified, all RGRCM variants in this section use the full training objective described in Section 3.4.

#### 4.5.1. Effect of the Proposed Components

Table 2 reports the contribution of the proposed components starting from the SATNet baseline. Adding the dedicated depth branch already provides a substantial gain, improving IoU from 80.69 to 83.02 and reducing BER from 7.33 to 6.72. This verifies that hierarchical sensor-depth features provide useful geometric cues when injected into the symmetry-aware decoder.

Adding RGRCM on top of the depth branch brings further improvements. In the “simple depth evidence” variant, DDEB is removed and the projected feature from Stage 2 of the depth branch is directly fed to RGRCM. Inside RGRCM, this feature replaces the DDEB output and is used as the input to both DRH and RGH. Even with this simplified design, the model improves IoU to 83.41 and BER to 6.48, showing that reliability-guided residual correction is effective when conditioned on an intermediate depth-branch feature. The full RGRCM, which replaces this simple feature with the complete DDEB representation, achieves the best overall performance with 83.57 IoU, 0.899 $F_{\beta }$, 0.026 MAE, and 6.26 BER. Although the difference in $F_{\beta }$ between the simple and full variants is marginal, the full design yields the best trade-off across all metrics, especially in MAE and BER.

**Figure 5:**
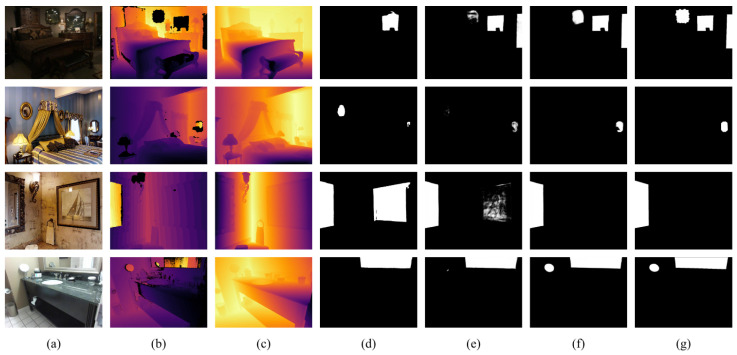
Qualitative ablation results of the proposed framework. From left to right, each column shows (**a**) input image, (**b**) sensor depth, (**c**) monocular predicted depth from Depth Anything v2, (**d**) SATNet, (**e**) SATNet + depth branch, (**f**) SATNet + depth branch + RGRCM (ours), and (**g**) ground-truth mask. The examples show representative challenging cases where sensor depth is corrupted, incomplete, or ambiguous around mirror regions. Compared with the RGB-only baseline, the depth branch recovers additional geometric cues but may still produce incomplete or noisy predictions. By further introducing RGRCM, the full model produces cleaner and more accurate masks that are more consistent with the ground truth.

Figure 5 provides a qualitative ablation analysis of the proposed components. Compared with the RGB-only SATNet baseline, adding the depth branch generally recovers additional mirror cues and improves the localization of challenging mirror regions. However, because the sensor depth is often corrupted or incomplete around reflective surfaces, the depth-branch-only variant may still produce noisy responses, miss weak mirror regions, or generate inaccurate shapes.

By further introducing RGRCM, the full model produces visibly cleaner and more complete predictions. In several examples, RGRCM suppresses spurious activations caused by unreliable sensor depth while preserving mirror regions that are weak in appearance or difficult to distinguish from surrounding structures. This behavior is consistent with the design of RGRCM, which uses dual-depth evidence and uncertainty-aware residual correction to refine the baseline prediction selectively rather than modifying all pixels indiscriminately.

The qualitative results therefore complement the quantitative ablation in Table 2. They show that the depth branch is effective for injecting geometric information into the decoder, while RGRCM further improves robustness by correcting difficult regions in a reliability-aware manner.

#### 4.5.2. Uncertainty Map Visualization

**Figure 6:**
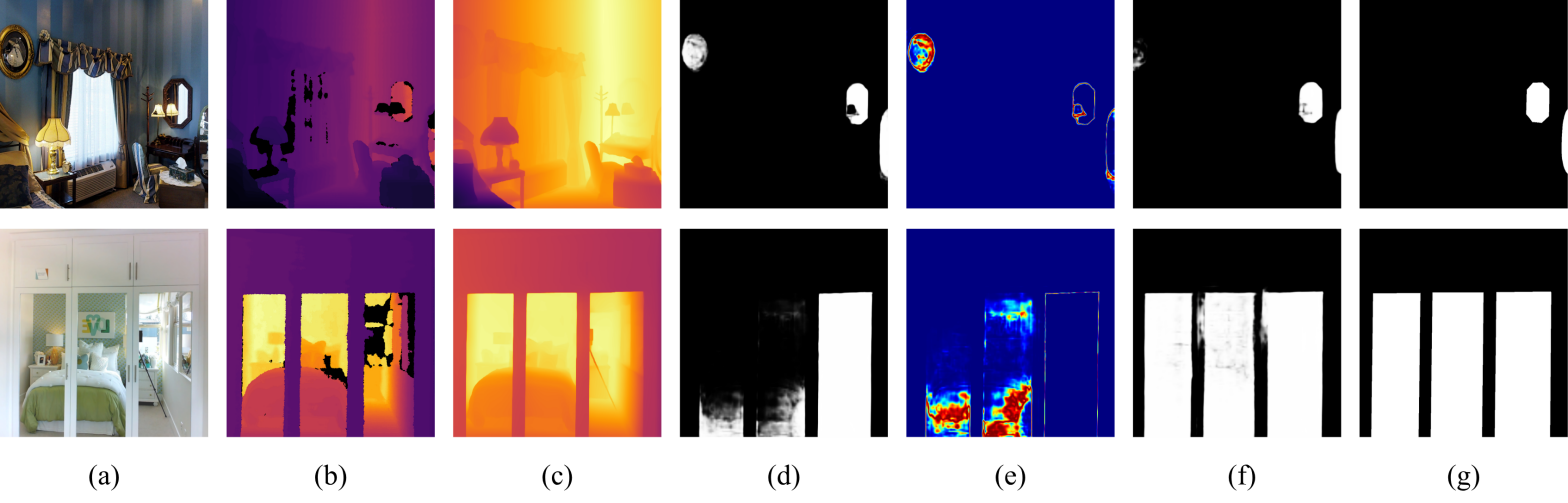
Visualization of the uncertainty map $H_{main}$ from RGRCM. From left to right: (**a**) input image, (**b**) sensor depth, (**c**) DA depth, (**d**) base prediction $P_{main}$, (**e**) uncertainty map $H_{main}$, (**f**) final prediction $P_{final}$, and (**g**) ground-truth mask.

Figure 6 visualizes the intermediate representations of RGRCM for representative test images. Each row shows, from left to right, the following: (a) the input RGB image, (b) the sensor depth map, (c) the monocular predicted depth from Depth Anything v2, (d) the base prediction $P_{main}$, (e) the uncertainty map $H_{main}$, (f) the final prediction $P_{final}$, and (g) the ground-truth mask.

The uncertainty map $H_{main}$ is computed as the normalized binary entropy of the base prediction $P_{main}$, where high values (warm colors) indicate regions of low prediction confidence. As shown in Figure 6, $H_{main}$ consistently highlights mirror boundaries and regions that share visual characteristics with mirrors, such as glass surfaces and reflective objects. In contrast, well-predicted background regions exhibit low uncertainty values (cool colors).

This spatial distribution of uncertainty directly governs the behavior of RGRCM through the final gate $G=H_{main}⊙G_{raw}$. In regions where $H_{main}$ is low, the gate is suppressed regardless of the learned reliability gate $G_{raw}$, preventing unnecessary depth-based correction from degrading already confident predictions. In regions where $H_{main}$ is high, the gate permits correction only when $G_{raw}$ additionally confirms that the dual-depth evidence supports reliable correction. This two-stage gating mechanism ensures that residual correction is applied selectively, focusing on ambiguous regions where depth-driven refinement is most likely to be beneficial.

#### 4.5.3. Statistical Stability

To verify the reliability of the reported improvements, we repeat the main ablation experiments with five different random seeds and report the mean ± standard deviation for all four evaluation metrics. In addition, we conduct paired *t*-tests between model variants using seed-matched runs on the same test set. While the main comparison tables report the primary run for consistency with prior single-run reports, this section provides additional multi-seed statistics to evaluate training stability.

Table 3 summarizes the results. The full model achieves 83.42±0.16 IoU, 0.898±0.002 $F_{\beta }$, 0.027±0.001 MAE, and 6.37±0.14 BER across five seeds. Compared with the RGB baseline, the improvement is statistically significant across all four metrics (p<0.01). Compared with the depth-branch-only model, the improvement in IoU is statistically significant (p=0.0294), while the improvements in $F_{\beta }$, MAE, and BER are directionally consistent but do not reach the 0.05 significance level. We report these non-significant results transparently to avoid overclaiming.

We do not compute *p*-values against competing methods because their repeated-run predictions are not publicly available. Computing paired statistics against single-run results from other groups would not be methodologically valid; we therefore restrict statistical testing to our own seed-matched ablation experiments. A further observation is that the standard deviation progressively decreases from the baseline (0.95) through the depth branch (0.25) to the full model (0.16). This trend suggests that the proposed modules not only improve mean performance but also reduce sensitivity to random initialization, contributing to more stable training.

#### 4.5.4. Robustness to Sensor-Depth Corruption

To evaluate how the proposed method behaves when sensor depth is degraded beyond the naturally occurring corruption in the dataset, we conduct a controlled robustness experiment. Three types of corruption are applied to the sensor depth at test time—without any retraining—and we compare the depth-branch-only model (without DDEB/RGRCM) against the full model to isolate the contribution of the proposed modules under degraded conditions.

The three corruption types are defined as follows. *Missing Depth* sets random pixels to zero at rates of 30%, 50%, and 70%, simulating the pixel-level sensor failure commonly observed near reflective surfaces. *Gaussian Noise* adds noise sampled from $N(0,\sigma^{2})$ with σ∈{3,5,10} in the 8-bit depth-intensity space (i.e., [0,255]), and it then converts the result back to [0,1] for the model, simulating electronic sensor noise and environmental interference. *Block Corruption* zeroes out contiguous 64×64 blocks at area ratios of 10%, 30%, and 50%, simulating regional sensor failure where an entire spatial neighborhood produces no valid measurement.

Table 4 reports the results. Across all three corruption types and all severity levels, the full model consistently exhibits a smaller IoU drop than the depth-branch-only baseline. For Missing Depth, the gap is most evident at 70% corruption, where the IoU drop decreases from 6.42 points (depth branch only) to 5.69 points (full model). For Gaussian Noise at σ=10, the IoU drop decreases from 1.49 to 0.90 points. For Block Corruption at a 50% area ratio, the drop decreases from 2.29 to 1.92 points.

These results indicate that the DDEB evidence channels—particularly the joint-validity channel $e_{7}$ and the local-variance channel $e_{8}$—enable the network to detect when sensor depth has become unreliable, while the RGRCM reliability gate suppresses correction in regions where depth information is uninformative. We note that these corruption experiments provide a controlled stress test within the RGBD-Mirror benchmark and do not replace evaluation on additional datasets, which we discuss as a limitation in Section 5.

#### 4.5.5. Effect of Sensor Depth, Predicted Depth, and Dual-Depth Evidence

Table 5 analyzes the roles of sensor depth, predicted depth, and the proposed dual-depth evidence. The first setting, *sensor depth only*, removes DDEB and uses only the sensor depth through the dedicated depth branch. This variant achieves 83.02 IoU and 6.72 BER, showing that valid sensor depth already provides strong geometric cues when injected into the decoder.

The second setting, *predicted depth only*, also removes DDEB, but it replaces the sensor depth input of the depth branch with the monocular predicted depth generated by Depth Anything v2. This variant achieves 82.50 IoU and 6.85 BER, which is lower than the sensor-depth-only setting. This result indicates that predicted depth can provide useful geometric information, but it is not a sufficient replacement for sensor depth in the depth branch.

The final setting uses the full model, in which the depth branch takes sensor depth as input and RGRCM internally constructs dual-depth evidence from both sensor depth and predicted depth through DDEB. This setting achieves the best performance across all metrics, reaching 83.57 IoU, 0.899 $F_{\beta }$, 0.026 MAE, and 6.26 BER. The comparison shows that sensor depth should remain the primary source for hierarchical geometric encoding, while predicted depth is most effective when used as a complementary cue for internal dual-depth evidence construction inside RGRCM rather than as a direct replacement for sensor depth.

We note that the primary value of DA depth in our framework is not as an independent input modality competing with sensor depth. Rather, it serves as a reference signal for assessing the reliability of sensor depth within DDEB. When sensor depth is corrupted, the discrepancy between sensor depth and DA depth provides an informative cue that DDEB encodes through its evidence channels. This interpretation is supported by the corruption robustness experiments in Table 4: under severe sensor-depth corruption (e.g., 70% missing depth), the full dual-depth model retains IoU 77.87 compared to 76.60 for the sensor-depth-only model. The benefit of dual-depth evidence becomes more pronounced precisely when sensor depth is most unreliable, which is consistent with the design rationale that predicted depth is most valuable as a reliability reference rather than a standalone geometric source.

#### 4.5.6. Effect of the Safe Loss Term

Table 6 evaluates the effect of the proposed safe loss term $L_{\mathrm{safe}}$ in the training objective of RGRCM. Without $L_{\mathrm{safe}}$, adding RGRCM on top of the depth branch does not improve IoU over the depth-branch baseline and yields only limited benefit in MAE, while the BER remains relatively high at 6.75. In contrast, when $L_{\mathrm{safe}}$ is included, the full model improves all evaluation metrics, reaching 83.57 IoU, 0.899 $F_{\beta }$, 0.026 MAE, and 6.26 BER.

This result shows that $L_{\mathrm{safe}}$ plays an important role in stabilizing the behavior of RGRCM. Since RGRCM performs residual correction on top of an already strong main prediction, unconstrained correction may unnecessarily perturb pixels that are already correct. The safe loss alleviates this issue by suppressing large corrections on pixels where the main prediction is already confident and consistent with the ground truth, thereby allowing the module to focus its correction capacity on ambiguous and difficult regions. As a result, the proposed loss improves not only BER but also IoU and MAE, confirming that it is an effective training objective for reliability-guided residual correction.

#### 4.5.7. Hyperparameter Sensitivity Analysis

Table 7 reports the sensitivity analysis for the two hyperparameters of the safe loss: the regularization weight λ and the uncertainty sensitivity α. For each experiment, one hyperparameter is varied while the other is fixed at its default value.

The choice of λ has a notable effect on performance. With λ=0.01, the regularization is too weak to prevent unnecessary corrections, yielding IoU 82.85 and BER 6.83. With λ=0.2, the residual correction is over-suppressed, and performance drops to IoU 83.04 and BER 6.70. The default value λ=0.1 achieves the best trade-off, reaching 83.57 IoU and 6.26 BER.

In contrast, α has a milder influence. Both α=5.0 and α=7.0 produce nearly identical results (IoU 83.57 vs. 83.56), while α=3.0 leads to a modest decrease (IoU 83.15). Since α controls how sharply the safe loss distinguishes confident predictions from uncertain ones, a value that is too small blurs this distinction and weakens the regularization effect. The results show that the default setting (λ=0.1, α=5.0) achieves the best performance among the tested settings and that the method is not overly sensitive to either hyperparameter within the tested range.

### 4.6. Complexity Analysis

Table 8 compares the computational complexity and accuracy–efficiency trade-off of the proposed method with representative RGB-D mirror segmentation methods under an input size of 416 × 416. PDNet is the lightest and fastest model in the table, achieving 153.8 FPS with 0.41 GB peak memory. However, its segmentation accuracy is substantially lower than that of the proposed method, with 77.77 IoU and 7.77 BER. In contrast, the proposed model requires more computation than PDNet but improves IoU by 5.80 points and reduces BER by 1.51 points, while still maintaining real-time inference at 57.8 FPS.

Compared with the SATNet baseline, adding the depth branch and RGRCM increases the computational cost only modestly, from 102.14 to 105.08 GFLOPs, and the parameter count from 125.35M to 126.13M. The final model achieves 57.8 FPS with 1.15 GB peak GPU memory. These results show that the proposed method does not aim to be the fastest model; rather, it provides a favorable trade-off between segmentation accuracy and computational efficiency. Note that, for PDNet, SATNet, and our variants, GFLOPs, FPS, and peak GPU memory were measured under the same environment using an input size of 416 × 416 and batch size 1. For reference, Table 8 also includes UTLNet, a recent RGB-D mirror segmentation method. Since the UTLNet FPS is reported from the original paper and was measured under a different hardware and implementation environment, it is used only as a contextual reference rather than a strictly direct speed comparison.

### 4.7. Failure Case Analysis

**Figure 7:**
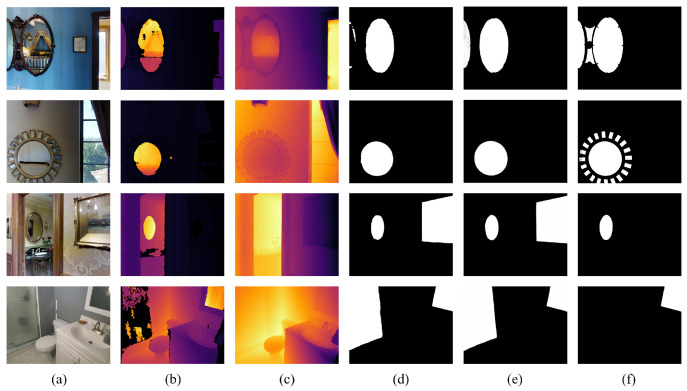
Representative failure cases of the proposed method. From left to right: (**a**) input image, (**b**) sensor depth, (**c**) DA depth, (**d**) SATNet (baseline), (**e**) our prediction, and (**f**) ground-truth mask. Rows 1 and 2 show unusual mirror shapes that deviate predominant in the training set. Row 3 shows a case with excessive missing depth, where the evidence channels carry insufficient information. Row 4 shows a glass surface misidentified as a mirror due to similar depth characteristics.

To provide a balanced evaluation, we analyze representative failure cases of the proposed method, as shown in Figure 7. We identify three failure modes. First, mirrors with irregular or uncommon shapes that deviate from the rectangular forms predominant in the training set can lead to incomplete segmentation boundaries. Second, when sensor depth contains an extremely high proportion of missing values, the dual-depth evidence channels carry little discriminative information, limiting the ability of DDEB to construct meaningful evidence for correction. Third, glass doors, windows, and other transparent or reflective surfaces may exhibit depth characteristics similar to those of mirrors, which can cause false positive predictions because depth-based evidence alone cannot fully distinguish mirrors from other reflective surfaces.

## 5. Conclusions

In this paper, we presented a reliable RGB-D mirror segmentation framework built upon SATNet. The proposed method extends the symmetry-aware RGB baseline with two key components: a dedicated depth branch for hierarchical sensor-depth encoding and a Reliability-Guided Residual Correction Module (RGRCM) for final prediction refinement. A central design choice of the proposed framework is that the predicted monocular depth is not used as an independent modality branch. Instead, it is combined with the sensor depth inside RGRCM to construct discrepancy-aware dual-depth evidence through the Dual-Depth Evidence Block (DDEB). This evidence is then used to support uncertainty-aware residual correction only where such correction is likely to be reliable and beneficial.

Extensive experiments on the RGBD-Mirror benchmark showed that the proposed design is effective. The depth branch consistently strengthened the SATNet baseline by providing explicit geometric cues, while RGRCM further improved the final prediction through reliability-guided residual correction. The full model achieved the best overall performance among the compared methods, reaching 83.57 IoU, 0.899 $F_{\beta }$, 0.026 MAE, and 6.26 BER. The ablation results further confirmed that sensor depth remains the primary geometric cue, while monocular predicted depth serves as a complementary source of internal evidence for more reliable correction.

Overall, the results suggest that the key to effective RGB-D mirror segmentation is not simply to add depth information, but to use it in a reliability-aware manner. By combining hierarchical sensor-depth encoding with selective dual-depth-guided correction, the proposed method provides a practical and effective solution for reliability-aware RGB-D mirror segmentation. We note that the current evaluation is limited to the RGBD-Mirror benchmark. Evaluation on additional RGB-D mirror segmentation datasets, when they become available, would further strengthen the generality of the proposed framework. The corruption robustness experiments demonstrate that the proposed method degrades more gracefully than the depth-branch-only baseline under the tested sensor-depth corruption conditions, providing evidence of robustness within the scope of this benchmark. In future work, it would be interesting to explore stronger hard-case modeling for severe depth corruption, finer boundary-aware correction strategies, and broader generalization to other reflective or transparent objects.

## Figures and Tables

**Table 1:** Comparison results of the mirror segmentation methods on the RGBD-Mirror benchmark. Best results are shown in bold. The results of Kurohiji and Hachiya ^†^ are taken from their original paper, where the reported values are the mean results of the top five runs selected from ten repeated trials. “–” indicates that the corresponding metric was not reported in the original paper.

Type	Method	IoU↑	$F_{\beta }$↑	MAE↓	BER↓
RGB mirror methods	VCNet	73.01	0.849	0.052	10.42
	SATNet	80.69	0.877	0.030	7.33
	CSFwinformer-B	78.66	0.863	0.031	8.57
	DPRNet	76.10	0.811	0.047	–
	S2MD	78.60	0.866	0.030	–
	SAMirror	79.20	0.836	**0.026**	10.02
RGB-D mirror methods	PDNet	77.77	0.825	0.042	7.77
	SANet	78.43	0.834	0.041	8.16
	NDANet-S*	79.93	0.844	0.035	7.56
	MGNet-S*	80.80	0.859	0.030	7.39
	UTLNet	80.50	0.858	0.032	7.23
	ADRNet-S*	82.21	0.871	0.030	7.02
	Kurohiji and Hachiya ^†^	70.94	0.881	0.079	–
	**Ours**	**83.57**	**0.899**	**0.026**	**6.26**

**Table 2:** Ablation results for the effect of the proposed components. The “simple depth evidence” variant removes DDEB and instead feeds the projected feature from Stage 2 of the depth branch into RGRCM. Specifically, this projected feature replaces the DDEB output as the input to both DRH and RGH. The “full” variant uses the complete DDEB representation inside RGRCM. The best result in each column is shown in bold.

Method	IoU↑	$F_{\beta }$↑	MAE↓	BER↓
Baseline (SATNet)	80.69	0.877	0.030	7.33
SATNet + Depth Branch	83.02	0.898	0.028	6.72
SATNet + Depth Branch + RGRCM (simple depth evidence)	83.41	**0.900**	0.028	6.48
SATNet + Depth Branch + RGRCM (full)	**83.57**	0.899	**0.026**	**6.26**

**Table 3:** Statistical stability over 5 random seeds with paired *t*-test *p*-values. The best result in each column is shown in bold.

Method	IoU↑	$F_{\beta }$↑	MAE↓	BER↓
Baseline (SATNet)	80.13 ± 0.95	0.875 ± 0.006	0.032 ± 0.002	7.84 ± 0.49
+ Depth Branch	83.04 ± 0.25	0.897 ± 0.003	0.028 ± 0.001	6.64 ± 0.20
+ DDEB + RGRCM (Full)	**83.42 ± 0.16**	**0.898 ± 0.002**	**0.027 ± 0.001**	**6.37 ± 0.14**
*Paired *t*-test *p*-values vs. Full model:*				
Baseline (SATNet)	0.0015 **	0.0006 ***	0.0053 **	0.0038 **
+ Depth Branch	0.0294 *	0.8712	0.0993	0.0695

*** p<0.001, ** p<0.01, * p<0.05.

**Table 4:** Robustness to sensor-depth corruption. Δ denotes the IoU drop from the clean baseline.

Method	Corruption	IoU↑	$F_{\beta }$↑	MAE↓	BER↓
SATNet + Depth Branch	No corruption	83.02	0.898	0.028	6.72
	Missing 30%	77.79 (−5.23)	0.859	0.033	9.97
	Missing 50%	76.71 (−6.31)	0.851	0.034	10.60
	Missing 70%	76.60 (−6.42)	0.851	0.034	10.69
	Noise σ = 3	82.68 (−0.34)	0.896	0.029	6.95
	Noise σ = 5	82.32 (−0.70)	0.893	0.029	7.16
	Noise σ = 10	81.53 (−1.49)	0.888	0.030	7.67
	Block 10%	82.47 (−0.55)	0.893	0.028	6.76
	Block 30%	81.37 (−1.65)	0.884	0.029	6.97
	Block 50%	80.73 (−2.29)	0.879	0.029	7.14
Ours	No corruption	83.57	0.899	0.026	6.26
	Missing 30%	78.94 (−4.63)	0.868	0.031	9.37
	Missing 50%	77.93 (−5.63)	0.861	0.032	9.98
	Missing 70%	77.87 (−5.69)	0.861	0.032	10.04
	Noise σ = 3	83.32 (−0.24)	0.899	0.027	6.45
	Noise σ = 5	83.11 (−0.46)	0.898	0.027	6.58
	Noise σ = 10	82.66 (−0.90)	0.896	0.028	6.89
	Block 10%	83.21 (−0.35)	0.897	0.027	6.41
	Block 30%	82.22 (−1.35)	0.889	0.028	6.85
	Block 50%	81.64 (−1.92)	0.886	0.029	7.14

**Table 5:** Effect of sensor depth, predicted depth, and dual-depth evidence. The first two settings remove DDEB, while the last setting uses the full model with DDEB inside RGRCM. The best result in each column is shown in bold.

Method	IoU↑	$F_{\beta }$↑	MAE↓	BER↓
Sensor depth only (without DDEB)	83.02	0.898	0.028	6.72
Predicted depth only (without DDEB; predicted depth as depth-branch input)	82.50	0.882	0.029	6.85
Sensor + predicted depth (full model with DDEB)	**83.57**	**0.899**	**0.026**	**6.26**

**Table 6:** Effect of the safe loss term in the proposed loss function. The best result in each column is shown in bold.

Method	IoU↑	$F_{\beta }$↑	MAE↓	BER↓
SATNet + Depth Branch + RGRCM (without $L_{\mathrm{safe}}$)	83.02	0.897	0.027	6.75
SATNet + Depth Branch + RGRCM (with $L_{\mathrm{safe}}$)	**83.57**	**0.899**	**0.026**	**6.26**

**Table 7:** Sensitivity analysis of safe loss hyperparameters λ and α. The best result in each column is shown in bold.

λ	α	IoU↑	$F_{\beta }$↑	MAE↓	BER↓
0.01	5.0	82.85	0.894	0.027	6.83
**0.1**	**5.0**	**83.57**	**0.899**	**0.026**	**6.26**
0.2	5.0	83.04	0.896	0.027	6.70
0.1	3.0	83.15	0.895	0.027	6.60
**0.1**	**5.0**	**83.57**	**0.899**	**0.026**	**6.26**
0.1	7.0	83.56	0.899	0.027	6.34

**Table 8:** Complexity comparison with representative methods.

Method	Input Size	GFLOPs↓	Params (M)↓	FPS↑	Peak Mem (GB)↓	IoU↑	BER↓
PDNet	416×416	82.31	80.54	153.8	0.41	77.77	7.77
UTLNet	416×416	157.74	263.69	9.5	-	80.50	7.23
SATNet (baseline)	416×416	102.14	125.35	60.8	0.59	80.69	7.33
+ Depth Branch	416×416	103.11	126.01	59.5	0.62	83.02	6.72
+ Depth Branch + RGRCM (ours)	416×416	105.08	126.13	57.8	1.15	83.57	6.26

## Data Availability

No new data were created in this study. The RGBD-Mirror dataset used in this work is publicly available and was introduced in reference [2]. Further inquiries can be directed to the corresponding author.
